# Supplementary material for: The magnitude of chronic diabetes complications and its associated factors among diabetic patients attending the general hospitals in Tigray region, Northern Ethiopia
Source: PLoS One. 2023 Aug 25;18(8):e0290240. doi: 10.1371/journal.pone.0290240 (PMC10456170; doi:10.1371/journal.pone.0290240)
Supplement: Attachment — Submitted filename: Response to reviewer.docx [file pone.0290240.s002.docx]

**Response to reviewer**

**Title page**

1. Research Title was modified as per your suggestion **: *“Magnitude of Chronic diabetes complication and its associated factors among adults with type 2 diabetes patients with type 2 diabetes mellitus in Tigray region, northern Ethiopia”…………………………………………………………..page-1)***

**Abstract**

2. Revised and paraphrased based on your comment : **“with age >60 years old [AOR, 3.00 (95% CI: 1.73, 5.26)]”** and all odds result were modified as per your comment……………………………(page-2)
3. DM prevalence in Ethiopia was revised using latest reports of IDF(20021)………………………(page-2)
4. The research gap statements in abstract section is revised as per your comment ………………...(page-2)
5. The method section of the abstract grossly revised /modified and types of data were explained --(page-2)
6. The group category of Duration of diabetes is modified as <5 or >5 based on previous literature and consistent with the table “diabetes duration of > 5 years **[AOR, 1.56 (95% CI: 1.18, 2.05)]”**……….. ..(page-2)

**Background**

7. The citation problem is corrected as it is replaced by latest (2021) IDF report for DM prevalence globally, in Africa and Ethiopia……………………………………………………………………..(Page3
8. DM prevalence in Ethiopia was revised using latest reports of IDF(20021)………………………(page-3)
9. The statement is already removed as the information is outdated and the Prevalence of DM in Ethiopia replaced by the latest report (2021) of IDF…………………………………………………………(page4)
10. The gab (methodological limitations) in the previous studies is clearly stated based on your study…………………………………………………………………………………………………(page4)

**Method**

11. **Study area & period**: Corrected as “ Tigray is”…………………………………………………(page4)
12. **Source population**: corrected based on your comment to include those who were not attend the hospital in data collection period he time frame of the data collection ………………………….(page 5)
13. **Eligibility:** As the study participant of this study was adults, the lower age limit for adulthood in Ethiopia is 18 years and patients with Type 1 diabetes (usually occurred at age <18 years) are not eligible for this study. Practically at time of data collection, the required follow up duration was at least 1 year and participants with such duration already included, **it was by mistake which is not edited in the document as > 1 year**…………………………………………………………………..(page5)
14. **Sample size determination**: The sample size for second objective (risk factor) was calculated using the assumptions used for the first sample size calculation ………………………………………..(page5)
15. **Sampling procedure**: All 13 general hospitals were not included in the study due to logistic issue /budget constraint. The reason why referral hospitals were excluded in this study is that in Ethiopia almost all diabetes related studies and all studies of chronic diabetes complication were done at referral hospitals which are found in big cities and diabetes populations who have follow up in general hospitals usually neglected in such studies although they are big in number. Primary hospitals were not included because diabetes service was not available. Moreover, the diabetes care services at specialized hospital is more advanced than general hospital then patients attending in such hospital usually with greater risk for complication. For those reasons both hospitals were excluded from the study……………(page5)
16. **Sampling procedure**: Yes proportional allocation to sample size was applied; We thought that it is not relevant to include omit Schematic/ pictorial presentation of sampling technique from the document Then as per your request we already include as **figure1** with other documents………………(Page 5)
17. **Sampling procedure**: K value was calculated and already present in the Schematic/ pictorial presentation of sampling technique (Figure 1)…………………………………………………..(Page5)
18. **Sampling procedure:** we are not clear with your question but in the document it is stated as “*The first patient was selected randomly from the first three by a lottery method, and the next patient was selected every three interval until the required sample was attained”*……………………………………(page5)
19. Data collection technique: because of the data collectors are with **multilingual skill (knew that English, Tigrigna and Amharic)** and official language is Amharic, then they can translate Tigrigna to Amharic or English for those who cannot speak Tigrigna …………………………………………………….(page 6)
20. **Data management and analysis**: First we enter the data using SPSS due to SPSS is easy/friendly to use and have difficulty to export from Epi Info to SPSS, later on we realized that error might be introduced although we try to use error minimizing strategy such as double data entry but we reenter the data using Epi Info 7 and export to SPSS 20 for analysis……………………………………(page7)
21. **Data management and analysis**: Hosmer-Lemeshow goodness-of-fit static test was done and the result was P=0.0063 which is considered as good model………………………………………..(page7)
22. **Operational definitions**: In this study we consider the major type of chronic diabetes complications and the cited reference /literature only mentioned the listed on but skin infection, sexual dysfunction and mouth disorder also occurred due to diabetes ……………………………………………….(page7)

**Results**

23. The statements **rewrite/ paraphrase** as per your comment “Thirty four percent of the participants had age greater than 60 years with mean age of 55.9 (SD+ 11.9) years in which one out of ten participants were free of at least one chronic diabetes complication” and “Of the total participants 54.0% were males, 67% were married, 88.0% were Orthodox christen and 96.3% were with Tigrian ethnic background and in which 29.4%, 34.0%, 47.1% and 52.4% participants had chronic diabetes complication respectively”…………………………………………………………………(page 9)

**Discussion**

24. **M**agnitude of chronic diabetes complication 54.0 % (95% CI 51.35-57.10) was refined as **54.0 % (95%, CI: 51.35, 57.10**)…………………………………………………………………(page 11)
25. As per your comment that statement was rewrite in separate paragraph and started as “This finding is in line with study in northern Ethiopia”………………………………………………………(Page 11)
26. The results were written for each country separately ‘…………………………………(page 11)
27. In separate paragraph and started as” The result of this study is higher than in India (33%), Sudan (46%) and west Ethiopia (34%)”……………………………………………………………(page11)
28. As per your comment separate reason/justifications were written for each ( lower and higher comparison)…………………………………………………………………………………(page 11)
29. There is an error in writing the figure from Jimma, Ethiopia study which is 25% not 39% then this figure is similar/inline to our study because it is within the range value……………………(page12)
30. Based on your comment the argument was written separately and clearly stated………(page 12)
31. The arguments for both comparison are stated separately and additional justifications are included to make strong and clear…………………………………………………………………………..(Page 12)
32. As per your comment arguments of both comparison are incorporated…………………..(Page12
33. The argument/justifications are written separately and clearly as per your comment……..(Page 13)
34. corrected as “finding is almost similar with study”…………………………………………(page 13)
35. It is corrected as per your comment and rewrite as “ 19.17 %(95%CI: 16.90,-21.44)……(page 14)
36. Study limitation it is already modified and rephrased properly……………………………(page 14,15)
