# Supplementary material for: The magnitude of chronic diabetes complications and its associated factors among diabetic patients attending the general hospitals in Tigray region, Northern Ethiopia
Source: PLoS One. 2023 Aug 25;18(8):e0290240. doi: 10.1371/journal.pone.0290240 (PMC10456170; doi:10.1371/journal.pone.0290240)
Supplement: Attachment — Submitted filename: Response to reviewers.docx [file pone.0290240.s003.docx]

**RESPONSE TO REVIEWERS**

**_______________________________________________________________________________________________________**

**Response to Reviewer #1**

I. **Minor comments**
1. **“Tigray”** is used consistently instead of “Tigrai” in the title, author affiliation, study area, and elsewhere in the manuscript ……………………………………………………………………………………………….(Page all)
2. “north Ethiopia / northern Ethiopia” was changed to **“ Northern Ethiopia**”…………………………(Page all)
3. "an institutional-based study," was replaced by ***“a multi-center cross-sectional study”*…………….**(Page1,4)
4. It was corrected as **54%** from 54.0% and as **27%** from 27.0% and other similar issues corrected throughout the document as per your suggestion .................................................................................................(Page: 2, 8,9,11)
5. *Comma* was used for numbers with more than two digits ***(e.g. 60000–100 000 was edited as 60,000-100,000)***………...(page all)
6. Double full stops were removed………………………………………………………………………...(page all)
7. The mentioned Ethiopian birr (ETB) in the Monthly income status section(Tables 1,3) was changed in to US dollar based on average exchange rate of 2019…………………………………………………………(Page 9)
8. The formula for sample size calculation was removed and rewrote in sentence format as per your recommendation and checked using StatCalc for population survey via Epi Info 7.0 software………..(Page4,5)
9. The result of **Hosmer-Lemeshow goodness-of-fit test was removed from the method section** and placed at the result section (Factors associated with chronic diabetes complication & table3 foot note), ……….(Page11)
10. Result of response rate is included/added in the result section of the manuscript………………….........(Page 8)
11. The multi variable analysis result was re-wrote as per your recommendation……………………...(Page 11, 12)
12. In Abstract & result section P-value was removed and re-wrote as (AOR=0.48; 95% CI 0.26, 0.9), and the hyphen (-) was removed instead comma was use for writing the 95% CI……..............................(Page 2, 11,12)
13. In appropriately cited references were checked & corrected via endnote software ...………………(Page 18-21)
14. The questionnaire has four parts, then corrected as **“ it has four parts”…**………………….……….....(Page 5)

_______________________________________________________________________________________________

II. **Major comments**
1. **Rationale of the study**: revised and additional scientific arguments are included ………………(Page 2,3,4)
2. The term “Risk factor” was replaced by **“factors associated”** and used consistently throughout the manuscript ………………………………………………………………………………………………(Page all)
3. **Sample size calculation**: …………………………………………………………………………...(Page 2.5,8)

The required sample size (n) was estimated manually using a single population proportion formula and cheeked using STATCALC for population survey via Epi-info version 7 software with assumptions of 95% CI (z = 1:96), d=0.03, and P=0.535.

Therefore the **initial sample size** was **1061.882 (** ni = (Z1-α/2)2p (1–p)/d2= (1.96)^2^0.535(1-0.535)/(0.03)^2^ )**.** However, a refusal rate of 10% (1061.882*0.1) **=106.1882** was added and gives a **final sample size of 1,168.0702 (1,061.882+106.1882),**

Accordingly10 questioners were excluded because of gross incompleteness and **1,158 participants’ questioner** were fit for final analysis which **makes response rate of 99.14 %** ………………………………

4. Incomplete sentences were re-wrote, spelling and grammar issues were checked and corrected…(Page all)

**_______________________________________________________________________________________________**

**___________________________________________________________________________________**

**Response to Reviewer #2:**

Ti**tle & abstract**

1. Gross English language Proofreading was done to correct errors of Spelling, grammar, punctuation and statement construction throughout the manuscript…………………………………………………. (Page all)
2. To avoid confusion with other similar abbreviations the expanded form of the abbreviations were included e.g. IDF (International Diabetes Federation ), Bsc is corrected as **BSc** and other abbreviations errors corrected accordingly ……………………………………………………………………………………..(Page 2,10,11 )
3. Research Title was modified as ***“Magnitude of chronic diabetes complication and its associated factors among diabetic patients attending the general hospitals in Tigray region, northern Ethiopia”*** *based on your suggestion* …………………………………………………………………………………………………………(page-1)

________________________________________________________________________________________________

4. **Method and materials**
   4.1 **Study Area**:: the study was done at 10 general hospitals out of all 14 (not 13, it was written by mistake) general hospitals, all general hospitals were not included because of the four hospitals were exclude randomly due to budget constraint / logistic issue…………………………………………………...(Page4)
   4.2 **Sampling**: A reference (ref.no 21) for **P=0.535, (p=proportion of chronic diabetes complications)** was included and this figure is used to calculate the sample size which was done in 2015 which was before our study conducted (2019/20) and taken from a study done at FelegeHiwot referral hospital, Bahardar, Amhara region, Northwest Ethiopia…………………………………………………………………..(Page5)

As you mention, by chance the chronic diabetes complication proportion of the study done at FelegeHiwot referral hospital (P=0.535) which we use for sample size calculation is similar to our finding (P=0.54). This could occur because of similarity in socio-demographic characteristics, poor glycemic control as a result of poor adherence to diabetes self-management recommendations……………………… (Page5, 11)

Therefore, the finding of this study (overall magnitude of chronic diabetes complication was p=0.54 or 54% of the patients had at least one type of the complication…………………………………… (Page 11)

But both studies have difference in many things such as sample size (344 vs. 1,158), study facility (one tertiary hospital vs. ten general hospitals), study area (Amhara region vs. Tigray region) and study period (2015 Vs. 2019/20)……………………………………………………………………………….... (Page4-6)

________________________________________________________________________________________________

4.3 **Operational definition**:
    4.3.1 **Definition of Hypertension**: The reference was updated and replaced by the reference that you recommend “European Society of cardiology (ESC) guideline 2018” (ref.no 33, 34)…………. (Page7,8)
    4.3.2 **Definition of other chronic diabetes complications**: all definition/ diagnostic criteria of other chronic diabetes complications (operational definitions) were updated according to the updated guidelines or manuscript based on your suggestion……………………………………….(Page 7,8)
    4.3.3 **Data collection and measurement(BP):** the timing of BP measurement related to coffee consumption was revised as 1-2 hours (*Study indicated that after caffeinated beverage intake blood pressure changes occur within 30 minutes, peak in 1-2 hours, and may persist for more than 4 hours)* and reference also included ………………………………………................................................................... (Page 6)

**___________________________________________________________________________________________**

5. **Result**
   5.1 **Socio-demographic, Clinical and behavioral characteristics:**

In this section we try to explain the findings based **on the cross-tabulation analysis results** but as mentioned in your review

- In which 29.4%, 34.0%, 47.1% and 52.4% participants had chronic diabetes complication respectively”
- In which 42.7%, 10.0% and 13.0% had chronic diabetes complication respectively.
- In which 6.0%, 32.2%, 19.7% and 6.6% participants had at least one chronic diabetes complication respectively.

Those are findings of **overall chronic diabetes complication** in terms of **socio-demographic, clinical and behavioral characteristics in the cross-tabulation analysis result** **(Table 1,2)** but we observed such way of explanation may result in confusion for the reader, so to make it clear and simple we prefer to omit/ remove all **cross-tabulation findings of chronic diabetes complication** from the **text explanation of socio-demographic, clinical and behavioral characteristics section** but readers can get those findings from the Table 1 & 2 …………………………………………………………………………………...(Page8-11)

5.2 **Tables:** all the abbreviations related to each table were placed in the footnote…… (Page 9,10,11,13)

6. **Discussion:** Except English language Proofreading, revision was not done in this section because you mentioned as “The discussion is well written”
7. **Conclusion:** Revision was not done because you stated as “It highlights the main findings and supported by the study results”
8. **Reference: Reference 10:** was replaced with updated reference**, Reference 22:** was replaced with updated reference**.** Accordingly all references were updated as per your suggestion ………(Page 18-21)

_______________________________________________________________________________________________
