# Supplementary material for: The magnitude of chronic diabetes complications and its associated factors among diabetic patients attending the general hospitals in Tigray region, Northern Ethiopia
Source: PLoS One. 2023 Aug 25;18(8):e0290240. doi: 10.1371/journal.pone.0290240 (PMC10456170; doi:10.1371/journal.pone.0290240)
Supplement: Attachment — Submitted filename: Response to reviewers.docx [file pone.0290240.s004.docx]

**RESPONSE TO REVIEWERS**

**Response to Reviewer #2**

1. **Abstract**
   1.1 **Conclusion:** revision was made based on your comment and it is revised as ***“In this study, more than half participants had at least one chronic diabetes complication”*…………………………………....**(Page 2)
2. **Method**
   2.1 **The exclusion and inclusion criteria:** the inclusion criteria and exclusion criteria are separately written under eligibility criteria to avoid confusion………………………………………………………...(Page5)
   2.2 **Diagnosis of chronic diabetes complication**
       2.2.1 **Coronary artery disease (CAD):** corrected as per your suggestion……………………….......(Page 7
       2.2.2 **Peripheral Vascular disease:** as per your suggestion the more than 1.3 (>1.3) was deleted from the definition, ABI to less than 0.9 only is used ………………………………………………………..(Page7)
       2.2.3 **Neuropathy:** misnomer of loss of sensitivity in the definition replaced with “ hyposthesia or anesthesia” in lower and upper limbs & the work limp is corrected as limb……………………...(Page7)
3. **Results**
   3.1 The wrongly written the word yes in the column is corrected throughout the tables………(Page 9,10,12 )
   3.2 All abbreviations (SBP, DBP) were written consistently throughout the document ………...(Page 10-17)
4. **Conclusion:** corrected as “In this study, more than half participants had at least one chronic diabetes complication”…………………………………………………………………………………….........(Page15)
5. **Language editing**: Our Manuscript was copyedit for language usage, spelling, and grammar by Zainabu Karim Mohamed from MUHAS, Tanzania (email: [zainab.karim4@gmail.com](mailto:zainab.karim4@gmail.com)) and Prof. Lilian T. Mselle (email: [nakutz@yahoo.com](mailto:nakutz@yahoo.com) ) from MUHAS, Tanzania. Uploaded as **Supporting Information file 1** ……………………………………………………………………………………………………….(all page)
